# Optimizing age of bull at first use in relation to fertility of Murrah breeding bulls

**DOI:** 10.14202/vetworld.2015.518-522

**Published:** 2015-04-21

**Authors:** M. A. Mir, A. K. Chakravarty, A. K. Gupta, B. C. Naha, V. Jamuna, C. S. Patil, A. P. Singh

**Affiliations:** 1 Dairy Cattle Breeding Division, National Dairy Research Institute, Karnal, Haryana, India; 2 Division of Animal Genetics, Indian Veterinary Research Institute, Barielly, Uttar Pradesh, India; 3 Department of Animal Genetics and Breeding, Lala Lajpat Rai University of Veterinary and Animal Sciences, Hisar, Haryana, India

**Keywords:** age at first use, conception rate based on first A.I, Murrah bull, overall conception rate

## Abstract

**Aim:**

The aim of the present investigation was to optimize the age at first use (AAFU) of semen of Murrah breeding bulls, which will help in early selection of bulls under progeny testing program for improving the reproductive performance in the herd.

**Materials and Methods:**

The data on AAFU, conception rate based on first A.I. (CRFAI), overall conception rate (OCR), and birth weight (B.WT) of 57 Murrah bulls during 1993-2014 at NDRI center pertaining to 14 sets of Network Project on Buffalo Improvement at ICAR-National Dairy Research Institute, Karnal, Haryana, India were adjusted for significant environmental influences and subsequently analyzed. Simple and multiple regression models were used for prediction of CRFAI and OCR of Murrah breeding bulls. Comparative evaluation of three developed models (I-III) showed that Model III, having AAFU and B.WT, fulfill the accuracy of model as revealed by high coefficient of determination, low mean sum of squares due to error, low conceptual predictive value, and low Bayesian information criterion.

**Results:**

The results revealed that the average predicted CRFAI was highest (39.95%) at <3.5 years and lowest (34.87%) at >4.5 years of age at first A.I/use. Similarly, average predicted OCR was highest (41.05%) at <3.5 years and lowest (39.42%) at >4.5 years of age at first A.I/use of Murrah bulls.

**Conclusion:**

In organized herd under progeny testing program, Murrah bulls should be used at young age, i.e. prior to 3.5 years, which is expected to result in 5.08% better CRFAI and 1.63% better OCR in comparison to Murrah bulls used after 4.5 years of age.

## Introduction

India has the largest buffalo population (108.7 million) in the world [1]. About 63% of the world’s buffalo milk and 95% of buffalo milk in Asia is contributed by Indian buffaloes [2]. India ranks first in the world with milk production (132.4 million tonnes) and the buffaloes contribute about 51.1% in the total milk production of India [3]. With the availability of frozen semen, the demand for the best buffalo males is increasing [4]. The reproduction parameters in animals are more influenced by environment [5]. Fertility of a herd depends mainly on early sexual maturity, in-time heat detection, minimum breeding interval, and number of services per conception of animal. Though the fertility is difficult to be measured exactly, however, it is indicated by the number of services taken by a bull for making an animal become pregnant. Bull fertility is measured by the percentage of cycling females exposed to the bull and impregnated during a specific time period usually between 60 and 130 days post-partum for Murrah buffaloes [6]. Breeding of livestock is costly and time consuming, so it is desirable to have higher conception rate in the herd. The genetically superior bull as a producer of large quantities of normal fertile spermatozoa in breeding program is important [7]. The availability of semen at the earliest possible age from breeding bulls is not only economical, but also may increase productive life span and proving the bulls under progeny testing program [7]. Without proper evaluation indiscriminate use of breeding bulls poses a potential threat to the dairy industry as it may transmit undesirable traits besides contributing to poor fertility of the herd. It has been found that age of the service sire affects the conception rate (CR) in dairy cattle [8]. Age of the bull at the time of mating is one of the major factors to determine reproductive performance of bulls [9]. A bull’s highest fertility observed at around 2-4 years of age, once the bull attains more than 4 years of age there may be some decline in bull fertility, but this is not very noticeable untill a bull attains 5-6 years of age [10]. Optimizing age of breeding bulls in relation to higher fertility will help in selection and use of breeding bulls at the right age thereby improving the reproductive efficiency of the herd [9].

Keeping the above in view, an attempt was made to optimize the age at first use (AAFU) of Murrah bulls, which would facilitate not only the selection of bulls at the right age but also help in designing more effective breeding programs.

## Materials and Methods

### Ethical approval

The present study was approved by Institutional Animal Ethics Committee of National Dairy Research Institute.

### Place of study

Karnal is situated at an altitude of 235-252 meters (748 feet) above the mean sea level at 29.68°N latitude and 76.98°E longitude in Eastern zone of Haryana which comes under the Trans-Gangetic plain agro climatic zone of India. The climate that prevails is subtropical in nature. The temperature in summer months (April to June) ranges between 24°C and 44°C. Karnal experiences moderate rainfall in the months of July and lasts till September. Winters are extremely cold. The temperature ranges from 4°C to 32°C in winter months (October, November, December, and January). Each year was sub-classified into four major seasons *viz*., winter (December to March), summer (April to June), rainy (July to September), and autumn (October to November), depending on prevalent meteorological factors as recorded in Central Soil Salinity Research Institute, Karnal [11]. The study was conducted on records of 57 Murrah bulls maintained under 14 sets of Network Project on Buffalo improvement at NDRI center. On standardization and normalization of traits, the number of bulls remained in the analysis were 56 for AAFU and 57 for birth weight (B.WT). The traits under study were AAFU, CR based on first A.I. (CRFAI), overall CR (OCR), and B.WT.

### Statistical analysis

The data were classified into various sub-classes to analyze the effect of non-genetic factors as season and period of birth, parity and age of dam for B.WT, period and season of use, parity, stages of lactation and age of buffalo for AAFU and period and season of A.I, parity, stages of lactation and age of buffalo for CRFAI, and OCR of breeding bulls, respectively. Reproduction and growth traits of Murrah bulls were adjusted for significant non-genetic factors by using fixed linear models. Since the data were non-orthogonal, the least-squares technique suggested by [12] was used to estimate the effect of non-genetic factors.

The model considered for CRFAI and OCR of Murrah bulls was considered as,


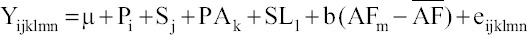


Where,

Y_ijklmn_=Observation on the n^th^ bull in i^th^ period of A.I., j^th^ season of A.I., k^th^ parity of buffalo, l^th^ stages of lactation of buffalo and m^th^ age of buffalo

m=Overall mean

P_i_=Effect of i^th^ period of A.I. (1-14)

S_j_=Effect of j^th^ season of A.I. (1-4)

PA_k_=Effect of k^th^ parity of buffalo (1-5)

SL_l_=Effect of l^th^ stage of lactation of buffalo (1-3)

b=Regression of age of buffalo on the CRFAI and OCR

AF_m_=Age of m^th^ buffalo


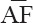
=Average age of buffaloes

e_ijklmn_=Random error ~ NID (0, σ^2^_e_)

The model for AAFU is considered as:

Where,

Y_ijklmn_=Observation on the n^th^ bull in i^th^ period of first use, j^th^ season of first use, k^th^ parity, l^th^ stages of lactation and m^th^ age of buffalo

m=Overall mean

P_i_=Fixed effect of i^th^ period of use (1-14)

S_j_=Fixed effect of j^th^ season of use (1-4)

PA_k_=Fixed effect of k^th^ parity (1-5)

SL_l_=Fixed effect of l^th^ stage of lactation (1-3)

b=Regression of age of buffalo on the trait

AF_m_=Age of m^th^ buffalo


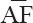
=Average age of buffaloes

e_ijklmn_=Random error ~ NID (0, σ^2^_e_)

The model considered for B.WT of Murrah bulls was considered as,


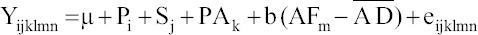


Where,

Y_ijkmn_ = Observation on the n^th^ bull in i^th^ period of birth, j^th^ season of birth, k^th^ parity of

dam and m^th^ age of dam

m=Overall mean

P_i_=Effect of i^th^ period of birth (1-14)

S_j_=Effect of j^th^ season of birth (1-4)

PA_k_=Effect of k^th^ parity of dam (1-5)

b=Regression of age of dam on B.WT of bulls

AD_m_=Age of m^th^ dam


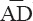
=Average age of dams

e_ijkmn_=Random error ~ NID (0, σ^2^_e_)

The difference of means between subclasses of periods, seasons, parity, and stage of lactation were tested for significance using Duncan’s Multiple Range Test [13]. The analysis of variance for season and period of freezing, stage of lactation, age of buffalo, and parity affecting different reproduction traits under model were computed.

### Model used for prediction of CR in Murrah bulls

Simple and multiple regression analysis were performed for prediction of CR using [14]. Three models were developed by using all possible combination of BW and AAFU for prediction of CR presented in Table-1. The coefficient of determination (R^2^) for each model is estimated and expressed in terms of percentage. Mallow’s Cp value is used for predicted model selection [15]. Akaike information criterion (AIC) as developed by [16] and Bayesian information criterion (BIC) as developed by [17] was estimated for model selection with different numbers of parameters. The model which have lowest AIC value, BIC value, Cp=<p, high R^2^, and minimum MSSe using all possible combination of B.WT and AAFU was judged as optimum model for prediction of CRFAI and OCR in Murrah bulls.

**Table-1:** Estimation of intercept and regression coefficients for prediction of CRFAI and OCR for Murrah bulls.

Traits	CRFAI			OCR		
	
	Intercept	Regression	Coefficients	Intercept	Regression	Coefficients
		b_1_	b_2_		b_1_	b_2_
AAFU	48.07	−2.8348	-	50.38	−3.1597	-
B.WT	13.03	0.7204	-	14.70	0.7393	-
AAFU, B.WT	24.12	−2.4805	0.6656	13.65	−2.2358	0.7428

AAFU=Age at first use, B.WT=Birth weight, CRFAI=Conception rate based on first A.I., OCR=Overall conception rate

### Optimization of age of bull at first use

Bulls were classified into three age groups *viz*.; <3.5 years, 3.5-4.5 years, and >4.5 years. The highest CR corresponding to the lowest AAFU of Murrah bulls was optimized by judging the predicted CR and average error of prediction in the respective age groups.

## Results and Discussion

B.WT of Murrah bulls was influenced by period of birth (p<0.01). AAFU of semen in Murrah bulls was found influenced by period (p<0.01) and season of use (p<0.05). CRFAI and OCR were found influenced by period and season of A.I. and age of buffalo (p<0.01) as presented in Table-2. The effect of parity and stage of lactation was found non-significant in all of the traits. Least-squares means of B.WT, AAFU, CRFAI and OCR estimated as 35.09±0.16 kg, 3.96±0.03 years, 40.27%, and 39.50%, respectively.

**Table-2:** Analysis of variance (M.S. values) of B.WT, AAFU, CRFAI and OCR of Murrah bulls.

Sources of variation	B.WT (kg)	AAFU (years)	CRFAI (%)	OCR (%)
Period of birth/use/A.I.	42.24** (13)	42.77** (13)	3136.70** (13)	3235.16** (13)
Season of birth/use/A.I.	2.13 (3)	1.92* (3)	384.59** (3)	389.78** (3)
Parity	23.44 (4)	0.35 (4)	92.36 (4)	78.71 (4)
Stage of lactation	-	0.49 (2)	1.72 (2)	2.58 (2)
Age of buffalo	13.86 (1)	0.31 (1)	235.86* (1)	694.31** (1)
Error	17.22 (35)	0.12 (32)	61.00 (4063)	55.31 (4063)

Figures in parentheses indicate respective degrees of freedom.

* p<0.05,

** p<0.01, B.WT=Birth weight, AAFU=Age at first use, CRFAI=Conception rate based on first A.I., OCR=Overall conception rate

## Optimizing age of bull at first use in relation to CR

The models for prediction of CRFAI and OCR of Murrah bulls have been developed using simple and multiple regression analysis. The intercept and regression coefficient of each model are presented in Table-1. For judging the optimum model for CRFAI and OCR of Murrah bulls various criterion values such as R^2^, MSSe, Cp, AIC, and BIC values for each model were estimated and presented in Tables-3 and 4. Looking into the judging of models it was observed that the Model III having B.WT and AAFU fulfilled four criterion like high R^2^, low MSSe, low Cp, and low BIC value.

**Table-3:** Estimation of criterion values, for judging optimum model for CRFAI in Murrah bulls.

Model number	Traits	P	R^2^	MSSe	Cp	AIC	BIC
I	AAFU	2	0.0791	0.2070	2.00	−40.10	−6.86
II	B.WT	2	0.1743	0.1856	2.00	−47.30	−8.67
III	AAFU, B.WT	3	0.2344	0.1670	3.00	−54.27	−8.83

P=Number of parameters, R^2^=Coefficient of determination, MSSe=Mean sum of square due to error, Cp=Conceptual predictive value, AIC=Akaike information criterion, BIC=Bayesian information criterion, CRFAI=Conception rate based on first A.I., AAFU=Age at first use, B.WT=Birth weight

**Table-4:** Estimation of criterion values, for judging optimum model for OCR in Murrah bulls.

Model no	Traits	P	R^2^	MSSe	Cp	AIC	BIC
I	AAFU	2	0.0833	0.1713	2.00	−52.60	−9.99
II	B.WT	2	0.1933	0.1580	2.00	−57.93	−7.42
III	AAFU, B.WT	3	0.2540	0.1315	3.00	−70.05	−12.77

P=Number of parameters, R^2^=Coefficient of determination, MSSe=Mean sum of square due to an error, Cp=Conceptual predictive value, AIC=Akaike information criterion, BIC=Bayesian information criterion, AAFU=Age at first use, B.WT=Birth weight, OCR=Overall conception rate

**Figure-1:**
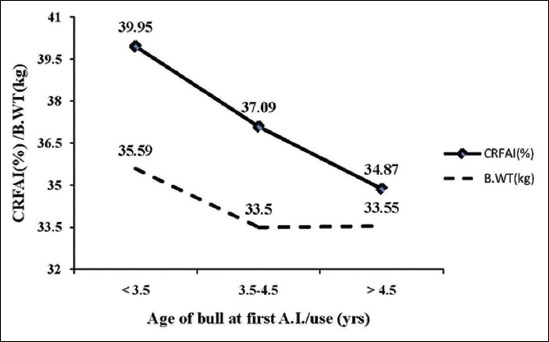
Optimum relation between birth weight, age at first use, and conception rate based on first A.I in Murrah bulls.

For optimizing AAFU in relation to fertility (CRFAI) of Murrah bulls, AAFU of Murrah bulls was classified into three groups i.e. <3.5 years, 3.5-4.5 years, and >4.5 years, presented in Table-5 and depicted in Figure-1. Average predicted CR in three age groups were estimated as 39.95%, 37.09%, 34.87% with the corresponding average B.WT of bulls were 35.59 kg, 33.50 kg, and 33.55 kg, respectively. Average predicted CR was found highest (39.95%) for Murrah bulls under <3.5 years, and lowest (34.87%) when AAFU of Murrah bulls was >4.5 years of age with average errors were 6.19% (<3.5 years), 15.81% (3.5-4.5 years), and 22.13% (>4.5 years), respectively.

**Table-5:** Optimum age at first use and predicted conception rate based on first and overall A.I. of Murrah bulls in relation to birth weight.

AAFU (years)	Number of bulls	B.WT (Kg)	CRFAI (%)	Average prediction error (%)	OCR (%)	Average prediction error (%)
<3.5	5	35.59	39.95	6.19	41.05	5.72
3.5-4.5	26	33.50	37.09	15.81	39.90	11.26
>4.5	7	33.55	34.87	22.13	39.42	7.67

B.WT=Birth weight, AAFU=Age at first use, CRFAI=Conception rate based on first A.I., OCR=Overall conception rate

**Figure-2:**
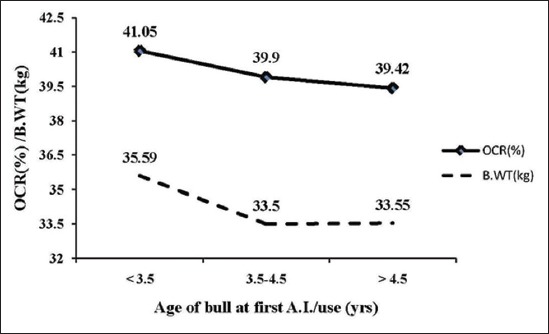
Optimum relation between birth weight, age at first use, and overall conception rate in Murrah bulls.

For optimizing AAFU in relation to fertility OCR of Murrah bulls, average predicted CR in three age groups were 41.05%, 39.90%, 39.42% and with the corresponding average B.WT of bulls depicted in Figure-2. Average predicted CR was found highest (41.05%) at <3.5 years and lowest (39.42%) at >4.5 years of AAFU of Murrah bulls with average errors were 5.72% (<3.5 years), 11.26% (3.5-4.5 years), and 7.67% (>4.5 years), respectively.

So far, research work has not been carried on optimization of AAFU of Murrah bulls in relation to better fertility in the herd. Many studies have reported that the probability of the bull having a desirable age influences the fertility in the herd. In India, buffalo bulls are put to service at about 3-3.5 years of age [7]. The best quality semen with regard to sperm morphology was observed in 3-5 year old Murrah bulls [18] and Nili-Ravi buffalo bulls [19]. Peak A.I. bull fertility has been reported at 2 years of age [20], whereas peak fertility at somewhat older ages of 3-4 years was also reported in dairy cattle [21]. 5 years age of the bull at the time of mating is the major factor with the variation of CR and fertility was found maximum, and the fertility started to decrease somewhat approximately up to age 9 or 10 years in Holstein bulls [9]. Several older studies on bull age were reviewed by Salisbury et al [22] and generally supported the pattern of results obtained by Kuhn, M.T. and Hutchison, J.L. [9].

In this research, “age of the bull” considered as the bulls age when it is used in the herd. Thus, bull age at insemination may have been 3.5-4.5 years, but semen collected when the bull was 2 or 2.5 years old, for example, may have been used in the insemination program. In spite of this caveat, results for bull age in Table-5 are generally consistent with the previous research and clearly showed marked improvement in predictions when bull AAFU was used in the predictor. Improved energy based feeding and management could possibly lead to further reduction in AAFU and improvement in predictions of CR of Murrah bulls. It is however necessary, the further study incorporating age of the bull at the time of collection, age of the bull first time used in the herd along with seminal parameters for assessing improved bulls fertility in the herd.

## Conclusion

The results revealed a negative association of CR with AAFU of Murrah bulls. To obtain maximum CRFAI and OCR of Murrah bulls, age of the bull at first A.I. should be <3.5 years under our management regime. Age of bull at A.I. was found to be a useful variable for improving the accuracy of predictions of bull’s CR in the herd. The findings of the present study will help in the early use of bulls thereby resulting in higher genetic and economic returns in a herd.

## Authors’ Contributions

AKC has planned the study. MAM recorded the information and analyzed the data. BCN, VJ, APS, and CSP provided help in the analysis of data. MAM drafted and revised the manuscript under the guidance of AKG and AKC. All authors read and approved the final manuscript.
